# Serum metabolomic profile as a means to distinguish stage of colorectal cancer

**DOI:** 10.1186/gm341

**Published:** 2012-05-14

**Authors:** Farshad Farshidfar, Aalim M Weljie, Karen Kopciuk, W Don Buie, Anthony MacLean, Elijah Dixon, Francis R Sutherland, Andrea Molckovsky, Hans J Vogel, Oliver F Bathe

**Affiliations:** 1 Department of Medical Sciences, 3330 Hospital Dr NW, Calgary, AB, Canada, T2N 4N1; 2 Department of Biological Sciences, University of Calgary, Biosciences Building, Calgary, AB, Canada, T2N 1N4; 3 Department of Mathematics and Statistics, University of Calgary, 2210 - 2nd St SW, Calgary, AB, Canada, T2S 3C3; 4 Department of Surgery, University of Calgary, 3330 Hospital Dr NW, Calgary, AB, Canada, T2N 4N1; 5 Department of Oncology, University of Calgary, Tom Baker Cancer Centre, 1331 - 29th St NW, Calgary, AB, Canada, T2N 4N2

## Abstract

**Background:**

Presently, colorectal cancer (CRC) is staged preoperatively by radiographic tests, and postoperatively by pathological evaluation of available surgical specimens. However, present staging methods do not accurately identify occult metastases. This has a direct effect on clinical management. Early identification of metastases isolated to the liver may enable surgical resection, whereas more disseminated disease may be best treated with palliative chemotherapy.

**Methods:**

Sera from 103 patients with colorectal adenocarcinoma treated at the same tertiary cancer center were analyzed by proton nuclear magnetic resonance (^1^H NMR) spectroscopy and gas chromatography-mass spectroscopy (GC-MS). Metabolic profiling was done using both supervised pattern recognition and orthogonal partial least squares-discriminant analysis (O-PLS-DA) of the most significant metabolites, which enables comparison of the whole sample spectrum between groups. The metabolomic profiles generated from each platform were compared between the following groups: locoregional CRC (N = 42); liver-only metastases (N = 45); and extrahepatic metastases (N = 25).

**Results:**

The serum metabolomic profile associated with locoregional CRC was distinct from that associated with liver-only metastases, based on ^1^H NMR spectroscopy (*P *= 5.10 × 10^-7^) and GC-MS (*P *= 1.79 × 10^-7^). Similarly, the serum metabolomic profile differed significantly between patients with liver-only metastases and with extrahepatic metastases. The change in metabolomic profile was most markedly demonstrated on GC-MS (*P *= 4.75 × 10^-5^).

**Conclusions:**

In CRC, the serum metabolomic profile changes markedly with metastasis, and site of disease also appears to affect the pattern of circulating metabolites. This novel observation may have clinical utility in enhancing staging accuracy and selecting patients for surgical or medical management. Additional studies are required to determine the sensitivity of this approach to detect subtle or occult metastatic disease.

## Background

While most individuals with metastatic colorectal cancer (CRC) receive treatments with palliative intent, there are some who may benefit from more aggressive surgical therapy with curative intent. The prototypical situation in which cure can still be achieved in the face of metastatic disease is when metastases are isolated to the liver. In patients with limited intrahepatic disease, and in the absence of extrahepatic disease, resection can result in a median survival of 40 to 58 months and a 5-year survival of 40 to 58% [1-4]. Presently, only 25 to 30% of patients with colorectal liver metastases have resectable disease. It is possible that earlier identification of the presence of liver metastases could increase the proportion of patients who could undergo surgery with curative intent. Therefore, biomarkers that facilitate early detection of liver-only metastases could be useful. In addition, biomarkers that reveal the presence of radiographically occult extra-hepatic disease could help to better select patients who would benefit from resection of liver metastases.

Biomarkers may be defined as any biomolecule or panel of biomolecules that can aid in the diagnosis of disease, prognostication, prediction of biology, or prediction of sensitivity to specific therapies. Recent biomarker discovery efforts have focused largely on the genome, the transcriptome and the proteome, using technologies that enable quantification of multiple biomolecules at once. In metabolomics, the biomarkers of interest consist of metabolites, small molecules that are intermediates, and products of metabolism, including molecules associated with energy storage and utilization, precursors to proteins and carbohydrates, regulators of gene expression, and signaling molecules. Thus, like the proteome, the metabolome represents a functional portrait of the cell or the organism. One potential advantage of metabolomics over proteomics is that metabolic changes may be more closely related to the immediate (patho)physiologic state of the individual. Relatively few biomarker discovery efforts have focused on the metabolome to date.

Our objective was to determine if, in patients with CRC, the serum metabolomic profile could be used to discriminate locoregional CRC from metastatic CRC, and to identify patients with liver-only metastases. We used proton nuclear magnetic resonance (^1^H NMR) spectroscopy because it is a well-established, robust and highly reproducible tool for obtaining a quantitative metabolomic profile of higher abundance metabolites. Gas chromatography-mass spectroscopy (GC-MS) was used to provide a more comprehensive metabolomic profile, and because it is a highly sensitive, rapid and accurate instrument for the detection of lower abundance metabolites. Using a combination of ^1^H NMR spectroscopy and GC-MS to obtain a relatively comprehensive metabolomic characterization, we determined that patients with locoregional CRC, liver-only metastases, and extrahepatic metastases could be discriminated using each of these approaches.

## Materials and methods

### Sample collection

This study was approved by the Conjoint Health Research Ethics Board at the University of Calgary (Ethics ID E21805). The study conduct conforms to the Helsinki Declaration. Clinically annotated serum samples were collected from consented patients who underwent surgery for resection of their primary colorectal adenocarcinoma, resection of liver metastases, or resection of extrahepatic metastases. All patients were treated at the Foothills Medical Centre, a tertiary referral centre, between 2004 and 2009. Patients with any acute inflammation or sepsis were specifically excluded. Surgical pathology was reviewed for all patients, and confirmed all had colorectal adenocarcinoma. Samples were collected in a plastic gold top Vacutainer tube (BD Biosciences, Mississauga, Ontario, Canada), which contained a clot activator and a gel for serum separation. Samples were processed within 6 hours of collection, then frozen at -20°C until the time of analysis. All samples were collected from patients who had fasted, prior to surgery.

### ^1^H NMR spectrometry

^1^H NMR spectroscopy was performed as previously described [5]. Briefly, all experiments were performed on a Bruker Avance 600 NMR spectrometer (Bruker Biospin, Milton, Canada) operating at 600.22 MHz and equipped with a 5 mm TXI probe at 298 K. One-dimensional ^1^H NMR spectra were obtained using a standard Bruker pulse sequence program (Bruker pr1d_noesy). Spectra were acquired as series of 1,024 scans, and then Fourier transformed using the Chenomx NMRSuite processor module in 65,536 data-points over spectral width of 7,211 Hz. A line broadening of 0.5 Hz was applied to all spectra before a standard procedure of phasing, B-spline baseline correction, water deletion, and reference deconvolution with DSS peak calibration using the Chenomx NMRSuite processor module. Metabolites were assigned based on comparison of both ^1^H and ^13^C chemical shifts and spin-spin coupling constants with those of model compounds in the Human Metabolome Database (HMDB, version 2.5) [6] and Chenomx NMR Suite 6.1 software (Chenomx Inc., Edmonton, Canada). Metabolites were quantified using the targeted profiling approach [7] as implemented in the Chenomx software. Metabolite intensities were integrally normalized against the sum of the metabolites' intensities for each sample, to adjust for possible inter-sample concentration variations.

### GC-MS spectrometry

The methods of Bligh and Dyer [8] were used for metabolite extraction. Briefly, layers of the two-phase mixture of chloroform and methanol were transferred to individual tubes. Aqueous layer tubes were dried under vacuum (SpeedVac, Eppendorf, Germany) and stored at -20°C until derivatization. For metabolite derivatization, 50 μl of methoxyamine-hydrochloride in pyridine solution (20 mg/ml) was added to each tube. N-Methyl-N-(trimethylsilyl) trifluoroacetamide (MSTFA; Sigma-Aldrich, Canada (Oakville, Ontario, Canada)) was added as silylating agent. Samples were diluted with hexane, and tubes were centrifuged to remove any solid particles and micro-particles. Ultimately, 200 μl of supernatant were transferred to a GC-MS vial with glass inserts, in preparation for GC injection.

GC-MS was performed on an Agilent chromatograph 7890A (Agilent Technologies Canada Inc, Mississauga, Ontario, Canada) coupled with a Waters GCT mass spectrometer, using GC-TOF-MS methodology. MS was operated in a range of 50 to 800 *m/z*. Mass spectra were processed using Metabolite Detector software (version 2.06, Technische Universität Carolo-Wilhelmina zu Braunschweig, Braunschweig, Germany). For metabolite identification, the GOLM metabolite database [9] and NIST 2008 library [10] were used. Identified peak intensities were integrally normalized against the sum of the peak intensities for each sample as the last step in the data pre-processing.

### Data analysis

Patients were allocated to one of three groups, based on stage and site of disease. Descriptive statistics were utilized to characterize the groups, with unpaired *t*-tests with unequal variances assumed (Welch's *t*-test) used to compare means and Fisher's exact tests used to compare categorical variables. All tests of significance were two-sided and a *P*-value < 0.05 was considered *a priori *to represent statistical significance between groups of patients in these univariate analyses.

Normalized data were log transformed, centered and unit variance scaled, then analyzed using the SIMCA-P+ program (version 12.0, Umetrics AB, Umeå, Sweden). Pairwise comparisons between the three groups were carried out using the same modeling approach, separately for each metabolomic platform. First, a preliminary principal component analysis (PCA) model was carried out including up to three components per PCA. This was done primarily to identify the potential variables that could form distinct sample subsets and intrinsic patterns, and to detect potential outlier samples (Figure S1 in Additional file 1). Next, selection of potentially important metabolites was carried out using two sample *t*-tests that assumed unequal variances. A *P*-value threshold of 0.3 was used to select these potentially important metabolites for inclusion in the supervised orthogonal partial least squares discriminate analyses (O-PLS-DA). In previous work, we have demonstrated that using this filtering approach to reduce the number of metabolites to those that are potentially informative results in a high degree of concordance between *P*-values obtained from univariate comparisons and variable influence on projection (VIP) values in O-PLS-DA models [5,11].

Three performance measures were used to assess the O-PLS-DA models: CV-ANOVA for assessing their reliability; R^2^Y, which describes the explained fraction of variation by the non-orthogonal component for the group status variable; and Q^2^Y, which is a measure of the predictability of the model. This predicted fraction was determined through a seven-fold cross-validation during the model-building process by leaving a seventh of samples out of every round and then testing the model against the remaining portion of data. R^2^Y and Q^2^Y scores range between 0 and 1, where an R^2^Y score of 1 demonstrates that 100% of variance is explained by the model, and a Q^2^Y score closer to 1 indicates higher reliability of the prediction in the cross-validation procedure. The R^2 ^score is always larger than the Q^2 ^score, but an observed difference of more than 0.3 between the R^2 ^and Q^2 ^scores should be carefully examined. Potential confounders (age, gender, and chemotherapy within 3 months of sample collection) were examined for their importance in our multivariate regression models. Receiver-operating characteristic (ROC) curves were used to provide summaries of the predictive performance of constructed models using Metz-ROC (University of Chicago, IL, USA).

### Pathway analysis

Known differentially abundant and co-regulated components in GC-MS analysis (on supervised O-PLS-DA) were used for metabolite pathway analysis using Metaboanalyst (version 2.0) [12]. This web-based software enables identification of altered metabolic pathways from its extensive HMDB-derived collection of more than 70 pathways and metabolite libraries.

Network and pathway analyses were generated using the Ingenuity Pathways Analysis (IPA) program (Ingenuity^® ^Systems [13]). A dataset containing chemical KEGG identifiers of the same components was uploaded into the program, one for each comparison. Each identifier renders a pertinent metabolite in Ingenuity's Knowledge Base, generating a list of network-eligible metabolites. These metabolites were then projected onto Ingenuity's knowledge-based global metabolite network. Subsequently, networks of these eligible molecules were algorithmically generated by IPA, based on their connectivity using functional core analysis.

## Results

### Patients and demographics

Patients with pathologically confirmed CRC who were potential candidates for surgery were included in the analysis. Sera were collected under standard fasting conditions. Patients were assigned to three groups: locoregional CRC (stages II and III, group 1, N = 42); liver-only metastases (group 2, N = 45); and extrahepatic metastases (group 3, N = 25). All patients with locoregional CRC and with liver-only metastases underwent resection. Patients with extrahepatic metastases underwent various surgical procedures to remove or to debulk all grossly apparent disease.

The characteristics of each patient group are summarized in Table 1. There were a number of differences in the groups that were evaluated in the multivariate model analyses to determine the effects of these covariables. Patients in the groups with metastatic disease were significantly younger, on average, than patients with locoregional CRC (*P *= 0.004), but there was no significant difference in average age between patients with liver-only metastases and extrahepatic metastases. There was a higher proportion of males in group 2 compared to group 1, but groups 2 and 3 had similar gender distributions. Chemotherapy was more frequently given within 3 months of sample collection in patients with metastatic disease. However, there was no statistical difference in the proportion of patients who had chemotherapy in groups 2 and 3. All administered chemotherapy agents are listed in Table 1.

**Table 1:** Patient characteristics of each group

		Liver-only	Extra-hepatic	*P*		
				
	Locoregional CRC: group 1 (N = 42)	metastases: group 2 (N = 45)	metastases: group 3 (N = 25)	Group 1 versus group 2	Group 2 versus group 3	Locoregional CRC versus all stage IV
Age (years)	72 ± 11	67 ± 13	63 ± 13	0.05	0.19	0.004
Gender				0.06	0.50	0.39
Male	21 (50)	31 (69)	15 (60)			
Female	21 (50)	14 (31)	10 (40)			
Bowel prep	39 (100)	45 (100)	25 (100)	NS	NS	NS
Stage						
Stage II	21	0	0			
Stage III	21	0	0			
Stage IV	0	45	25			
Any chemotherapy within 3 months	5 (21)	16 (36)	9 (36)	0.02	0.79	0.02
Specific chemotherapeutic agents						
5-FU	5 (100)	15 (94)	7 (78)			
Oxaliplatin	1 (20)	7 (44)	4 (44)			
Irinotecan	0 (0)	10 (63)	2 (22)			
Bevacizumab	0 (0)	2 (13)	0 (0)			
Other chemotherapy	1 (20)	1 (6)	2 (22)			

Numbers in brackets represent percent (%). 5-FU, 5-fluorouracil.

To evaluate the effects of each of the potential confounders (age, gender, exposure to chemotherapy within 3 months) on metabolomic profiles, we developed O2-PLS-DA regression models that included the effects of these factors in these models. All regression models revealed that none of these factors had significant confounding effects on the metabolomic profiles and so were not included in the final O-PLS-DA models.

### Distinguishing locoregional CRC from liver-only metastases

**Figure 1:**
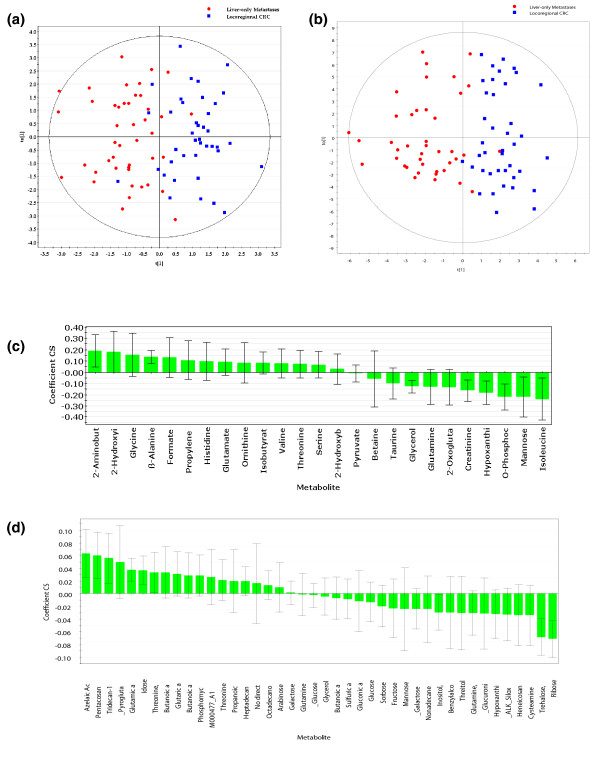
**Comparison of metabolomic profiles from patients with locoregional CRC and liver-only disease**. **(a) **O-PLS-DA scatter plot depicting metabolomic profiles analyzed by ^1^H NMR spectroscopy. **(b) **O-PLS-DA scatter plot depicting metabolomic profiles analyzed by GC-MS. **(c) **Coefficient plot demonstrating relative abundance of specific metabolites detected by ^1^H NMR spectroscopy. Metabolites on the left are more abundant in sera from patients with liver metastases, and metabolites on the right are most abundant in locoregional disease. **(d) **Coefficient plot demonstrating relative abundance of specific metabolites detected by GC-MS. Only identified metabolites are included. t[1], score for the predictive component in O-PLS-DA; to[1], score for the Y orthogonal component in O-PLS-DA.

By ^1^H NMR spectroscopy, 55 metabolites were detected, with 25 found to be differentially abundant in the initial data filtering process, using a *P*-value < 0.30. This cutoff was used to select only the potentially informative metabolites, to be included in subsequent supervised multivariate analysis (O-PLS-DA). By ^1^H NMR spectroscopy alone, there was a robust distinction between liver-only metastases and locoregional CRC (R^2^Y score = 0.61). The predictive ability of the model was measured by seven-fold cross-validation (Q^2 ^score = 0.39, CV-ANOVA *P*-value = 5.10 × 10^-7^; Figure 1a). The coefficient plot demonstrating degree of differential abundance for each metabolite is depicted in Figure 1c.

GC-MS could detect 476 components across the entire range of samples, of which 170 were identified as metabolites. We found 39 known metabolites and 114 unidentified components to be differentially abundant between patients with locoregional CRC and patients with liver-only metastases, using two sample *t*-tests with *P*-value cutoffs of 0.3. Following noise filtration, O-PLS-DA of the 124 remaining components demonstrated that patients with liver-only metastases could be distinguished from patients with locoregional disease (R^2 ^score = 0.68, Q^2 ^score = 0.40, CV-ANOVA *P*-value = 1.79 × 10^-7^; Figure 1b). The coefficient plot corresponding to the degree of differential abundance of each feature is shown in Figure 1d. Table 2 provides a list of identified metabolites found by each analytical modality to be differentially abundant between patients with locoregional CRC and liver-only metastases.

**Table 2:** Metabolites found to be differentially abundant in ^1^H NMR and GC-MS in pair of patient groups

Group comparison, analytical platform	Increased in liver-limited metastases		Decreased in liver-limited metastases	
	
	Metabolite	*P*	Metabolite	*P*
Liver-only disease versus locoregional disease, ^1^H NMR	2-Aminobutyrate	0.07	Isoleucine	0.13
	2-Hydroxyisovalerate	0.05	Mannose	0.10
	ß-Alanine	0.09	O-Phosphocholine	0.08
	Formate	0.005	Hypoxantine	0.10
	Histidine	0.16	Creatinine	0.11
	Glutamate	0.0007	2-Oxoglutarate	0.02
	Isobutyrate	0.002	Glutamine	0.0004
			Glycerol	0.05
Liver-only disease versus locoregional disease, GC-MS	Azelaic acid	0.11	Ribose	0.03
	Pentacosane	0.30	Trehalose	0.22
	Tridecan-1-ol	0.19	Cysteamine	0.15
	Pyroglutamate	0.13	Heneicosane	0.13
	Idose	0.04	Glutamine	0.10
			Benzyl alcohol	0.19
			Myo-inositol	0.23
			Nonadecane	0.13
			Galactose	0.03
			Mannose	0.02
Extrahepatic metastases versus liver-only disease, ^1^H-NMR	Isoleucine	0.03	Methionine	0.07
	2-Oxoglutarate	0.07	Fumarate	0.23
	Mannose	0.07	Tyrosine	0.24
	Glutamine	0.12	Serine	0.07
	Leucine	0.12	Formate	0.10
	2-Aminobutyrate	0.16	Alanine	0.17
			Glutamate	0.14
Extrahepatic metastases versus liver-only disease, GC-MS	Tridecan-1-ol	0.003	Butanoic acid, 3-hydroxy	0.12
	Sulfuric acid	0.25	Glutamine	0.14
	Pentadecan-1-ol	0.07	Glucuronic acid	0.05
	Phenylalanine	0.10	Myo-inositol	0.23
	Tetradecanoic acid	0.03	Uric acid	0.30
	Octadecadienoic acid	0.03	Glucose	0.16

Metabolites in these tables were selected based on the two-sample *t*-test statistics used for data filtering (*P*-value < 0.30) and the threshold of variable importance in the projection (VIP) > 0.8. Correlation with the model comparison is determined by scaled and centered coefficients.

We further analyzed the group with liver-only disease to derive information on the sensitivity of metabolomics-based tests for detection of liver metastases. Solitary metastases were present in 23 patients. These ranged in size from 14 to 99 mm in maximal diameter. Regression models revealed that number of liver lesions (solitary versus multiple) did not have significant confounding effects on the metabolomic profiles. Indeed, when only patients with solitary nodules were included, metabolomic profiles remained different in the two stage groupings, by ^1^H NMR spectroscopy (*P *= 2.60 × 10^-5^) and by GC-MS (*P *= 4.17 × 10^-5^).

To ensure that chemotherapy had no inadvertent effect on our ability to distinguish between locoregional disease and liver metastases, we excluded patients who had chemotherapy within 3 months of sample collection, and utilized the same models to compare these two groups. This confirmed that the metabolomic profiles were different in the two stage groupings, by ^1^H NMR spectroscopy (*P *= 5.32 × 10^-6^) and by GC-MS (*P *= 0.006).

### Distinguishing liver-only metastasis from extrahepatic metastasis

**Figure 2:**
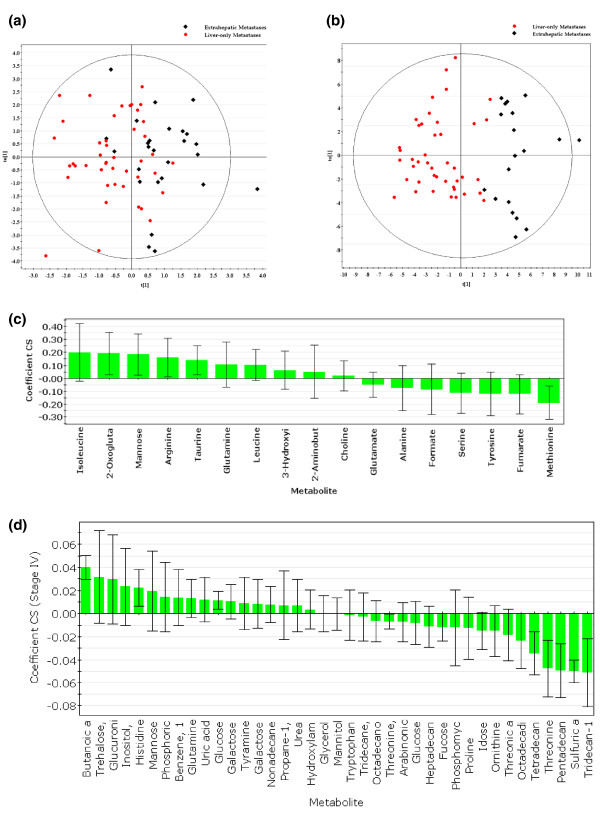
**Comparison of metabolomic profiles from patients with liver-only metastases and with extrahepatic metastases**. **(a) **O-PLS-DA scatter plot depicting metabolomic profiles analyzed by ^1^H NMR spectroscopy. **(b) **O-PLS-DA scatter plot depicting metabolomic profiles analyzed by GC-MS. **(c) **Coefficient plot demonstrating relative abundance of specific metabolites detected by ^1^H NMR spectroscopy. Metabolites on the left are more abundant in extrahepatic metastases, and metabolites on the right are most abundant in liver metastases. **(d) **Coefficient plot demonstrating relative abundance of specific metabolites detected by GC-MS. Only identified metabolites are included. t[1], score for the predictive component in O-PLS-DA; to[1], score for the Y orthogonal component in O-PLS-DA.

After statistical filtering using a *t*-test to remove uninformative metabolites, 17 metabolites were included in the regression analysis in ^1^H NMR profiling for the comparison of patients with liver-only metastases and patients with extrahepatic metastases. In this instance, orthogonal discriminant analysis did not produce the same strong discriminant components for distinguishing between these groups of patients as was found in the analysis between locoregional CRC and liver-only metastases. In this model, R^2^Y was only 0.36 and the model was not strongly predictive of metastatic site (Q^2^Y score = 0.13; CV-ANOVA *P*-value = 0.04; Figure 2a). Having said this, isoleucine and 2-oxoglutarate were more abundant in sera from patients with extrahepatic metastases, while methionine and fumarate were more abundant in liver-only metastases (Figure 2c and Table 2).

Interestingly, GC-MS was more capable of identifying differences between patients with liver-only metastases and extrahepatic metastases. After feature selection of the GC-MS data, 152 components were used for discrimination modeling between these two patient groups, of which 59 were identified as metabolites. The resulting model included metabolites that explained much of the variation in the groups (R^2^Y score = 0.69), and it was predictive (Q^2^Y score = 0.54; CV-ANOVA *P*-value = 4.75 × 10^-5^) (Figure 2b). Figure 2d depicts the contributions of each feature to the model, and Table 2 provides a list of identified metabolites that were seen to be differentially abundant.

Again, to ensure that chemotherapy did not inadvertently affect our observations, we used the same models in patients who had not been exposed to chemotherapy within 3 months of sample collection. This analysis confirmed that the metabolomic profiles continued to be different in the two patient groupings, by ^1^H NMR spectroscopy (*P *= 0.69) and by GC-MS (*P *= 3.78 × 10^-5^).

### Internal verification of clinical applicability

The ROC curve is an indicator of the predictive performance of a developed test and depicts the range of relationships between sensitivity and specificity. In this study, we tested the predictive performance of our discriminant models to distinguish between pairs of disease states (locoregional disease, liver-only metastases, and extrahepatic metastases) by constructing seven models with one-seventh of the data excluded from each model, and with each sample excluded once. The ability of the average of the seven models to predict the excluded samples provided a measure of the predictive ability of each metabolomic profiling model. Using these average predicted group values (Ypredcv from the Umetrics software), we were able to generate a ROC for each comparison.

**Figure 3:**
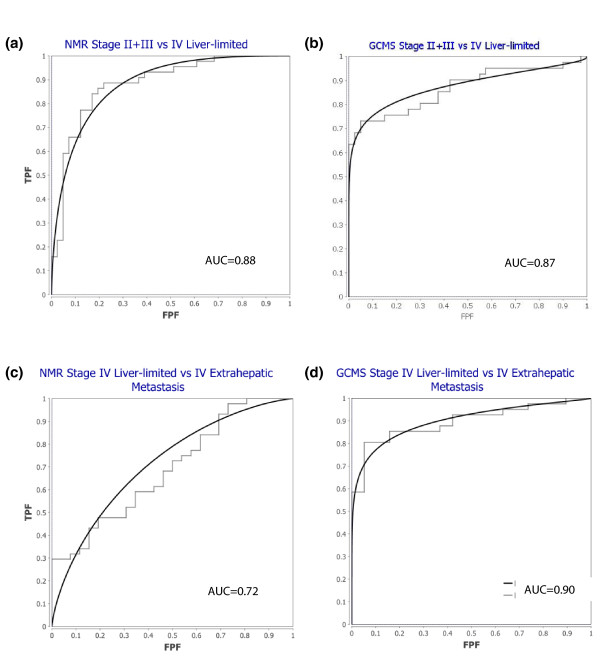
**ROC curves depicting the predictive performance of generated classifiers in each comparison**. **(a) **ROC curve illustrating performance of the NMR model in distinguishing liver-only metastases from locoregional CRC. **(b) **ROC curve illustrating performance of the GC-MS model in distinguishing liver-only metastases from locoregional CRC. **(c) **ROC curve for the NMR model distinguishing extrahepatic metastases from liver-only metastases. **(d) **ROC curve for the GC-MS model distinguishing extrahepatic metastases from liver-only metastases. AUC, area under the ROC curve; FPF, false positive fraction; TPF, true positive fraction.

ROC curves were plotted for ^1^H NMR spectroscopy and GC-MS to demonstrate the ability to predict the presence of liver-only metastases or locoregional CRC. The area under the ROC curve (AUROC) was 0.88 for ^1^H NMR spectroscopy and 0.87 for GC-MS (Figure 3a and 3b, respectively). Values greater than 0.8 indicate excellent predictive ability. The *P*-values for cross-validation in both series were remarkably low and indicate excellent predictive ability. These data demonstrate that the metabolomic profile can be useful to identify the presence of liver metastases or, at least, to distinguish patients with locoregional disease and liver-only metastases.

ROCs were also calculated for ^1^H NMR spectroscopy and GC-MS to demonstrate the ability to predict the presence of extrahepatic metastases. While the AUROC was only 0.72 for ^1^H NMR spectroscopy, it was still very high for GC-MS (AUROC 0.90) (Figure 3c and 3d, respectively), which may be attributed to the higher sensitivity of the MS analytical platform.

### Pathway analysis

We were intrigued that the metabolomic profile differed so dramatically in the sera of patients with locoregional disease as compared to liver-only metastases. Further analysis was conducted to glean some understanding of whether this was a reflection of differences in tumor biology, or due to differences in the host response to disease involving different organs, or both. Metabolomic pathway analysis and network analysis were performed using data derived from GC-MS.

Accelerated galactose metabolism was apparent (*P*-value = 0.0006 on univariate analysis). The liver is central to galactose metabolism; however, there are no reported alterations in galactose metabolism in tumor cells. Accelerated glutamine and glutamate metabolism was also apparent (*P*-value = 0.04 on univariate analysis). Again, the liver is known to actively take up glutamine and convert it to glutamate, making it available for gluconeogenesis or for subsequent conversion to other amino acids. Glutaminolysis is also known to be an important energy source in tumor cells, including in CRC [14-16].

**Figure 4:**
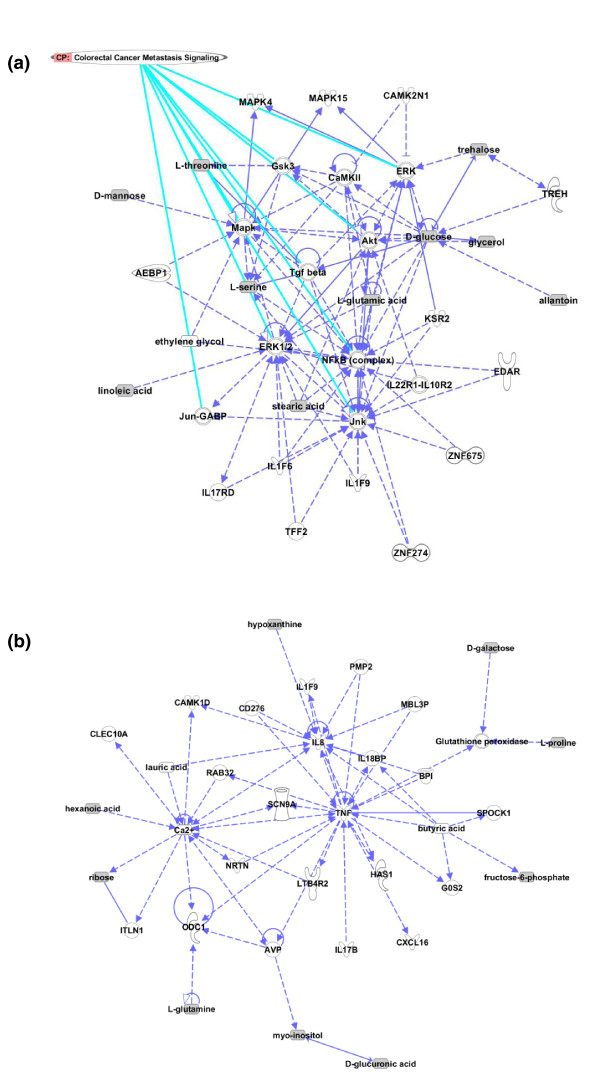
**Pathway analysis derived by comparison of the relative abundance of metabolites from sera derived from patients with locoregional CRC and liver-only metastases, as determined by GC-MS**. More centrally located molecules in the illustrated networks have a greater probability of participating in the biological processes involved in metastasis, but also represent hubs of diverse known biological functions. **(a) **The first network highlights the contribution of mediators of proliferation, apoptosis and energy consumption, as well as a prominent role of inflammatory mediators. As indicated, some of the molecules putatively involved are known for their contribution to the pathogenesis of metastasis in colorectal cancer. **(b) **The second network demonstrates that inflammatory processes are highly involved in the metastatic process.

A network analysis was performed to explore potential upstream altered pathways associated with liver metastases. The IPA network analysis uses information extracted from the literature to extrapolate known signaling and metabolic pathway relationships from the (co-related) metabolites found to be differentially abundant in our experiments. Two networks, representative of observed changes in levels of identified compounds, could be constructed. In the first network, higher levels of NFkB, mitogen-activated protein kinase (MAPK) and its related Ca^2+^/calmodulin-dependent protein kinase II (CaMKII) complex, JNK and ERK1/2 are predicted to be involved with liver metastasis (Figure 4a). Interestingly, this combination of signaling complexes and pathways typifies the colorectal cancer metastasis signaling pathway [17-24]. In this first network, there was also higher activity of several kinases and inflammatory cytokines in the context of liver metastasis. These have not previously been shown to have a direct contribution to metastasis of colorectal cancer. CaMkII, a kinase for several mediators in cell proliferation and apoptosis pathways, is one such molecule. In the second network, a highly connected web of inflammatory mediators, including TNF, IL-8, and IL-17B, could be visualized (Figure 4b). IL-17B was recently identified to activate both TNF and NFkB pathways [25]. IL-17B-induced expression of TNF and IL-1β results in monocytic chemotaxis [26], a phenomenon that is well described in colorectal liver metastases [27,28].

^1^H NMR spectroscopy data were then utilized for pathway analysis. Because fewer metabolites were found to be differentially abundant (compared to GC-MS), it was considered that using these data may not yield a particularly accurate picture of altered metabolic pathways. Remarkably, however, the network derived from pathway analysis using ^1^H NMR spectroscopy data revealed a role by many of the same signaling molecules and inflammatory mediators demonstrated by analysis of the GC-MS data (Figure S2 in Additional file 2).

We interpreted this analysis to reflect the fact that tumors that metastasize differ biologically from tumors that are confined to the colon. In addition, these data may reflect the response of liver to the local effects of tumor. This pathway analysis therefore supports the hypothesis that the metabolomic profile that distinguishes liver metastases from locoregional CRC reflects elements of a site-specific host response to tumor, as well as changes in tumor biology associated with metastasis.

## Discussion

Presently, preoperative staging for CRC involves radiographic studies such as CT scans to determine extent of disease. Operative findings and pathological examination of the surgical specimen(s) result in a modification of the initially assigned stage. Specifically, the depth of tumor invasion and involvement of lymph nodes are determined. In some cases, however, occult metastatic disease can be missed using contemporary staging methods. Postoperatively, patients are followed closely for local or distant recurrence, in hopes that early detection will hasten treatment before it becomes disseminated. The current guidelines by the American Society of Clinical Oncology suggest annual CT scans for patients eligible for curative surgery [29], as well as serum carcinoembryonic antigen (CEA) every 3 months for stage II and III disease for at least 3 years if the patient is a candidate for surgery or chemotherapy for metastatic disease [30]. This intensive postoperative follow-up is designed to detect metastatic disease that is amenable to resection. For example, limited liver metastases in the absence of extrahepatic disease may be resected. Biomarkers that facilitate the detection of occult metastatic disease before or after surgery would therefore enhance the staging of CRC patients, potentially impacting on treatment decisions.

Using ^1^H NMR spectroscopy and GC-MS, we have demonstrated convincingly using internal validation that the serum metabolomic profile differs in patients with locoregional CRC and metastatic CRC. Moreover, we have observed that there are differences in serum metabolomic profile between patients with metastatic disease that is confined to the liver and extrahepatic metastases. This is a novel finding. External validation will be required to confirm the exact metabolic alterations that occur with each disease state. In addition, more work will be required to determine the sensitivity of the changes. That is, it will be essential to determine the minimal amount of intrahepatic or extrahepatic disease that can be detected by this technique. In order for this biomarker approach to be clinically useful, it must be possible to detect even small, solitary liver metastases, and it must be possible to detect radiographically invisible extrahepatic metastases. Our data are promising in this regard, as a large proportion of patients in the liver-only disease group had solitary metastases as small as 14 mm. Finally, the unique and complementary roles of ^1^H NMR spectroscopy and GC-MS must be evaluated, for a test that is based on a single analytical modality may be more feasible and cost-effective than a test employing two analytical modalities.

Metabolomic biomarkers have numerous advantages over transcriptomic and proteomic biomarkers. First, changes in the metabolome are amplified relative to changes in the transcriptome and proteome [31]. Therefore, metabolites may change even when protein levels do not. Second, metabolomic profiling is cheaper and easier than proteomic and transcriptomic profiling. Thus, a test based on metabolomics could be more easily implemented in the clinic. Third, changes in metabolism result in alterations of the abundance of groups of metabolites. Therefore, identification of the patterns of changes in metabolites would provide insight into the functional changes that occur due to any given condition. The metabolomic profile therefore represents a complex biomarker of considerable interest, albeit one that has been studied relatively little.

There have been only four reports so far of serum metabolomic changes associated with CRC, and none have described stage- or organ-specific changes to the metabolomic profile. Qiu *et al. *[32] compared 64 Chinese patients with CRC to healthy controls; metabolomic profiles were determined by GC-MS and liquid chromatography-mass spectrometry (LC-MS). The metabolomic profiles in CRC patients (including eight patients with stage IV CRC) were distinct from those of healthy controls. Interestingly, several metabolites were differentially abundant in all stages of disease. This study demonstrated the feasibility of using metabolomics to diagnose CRC. Kondo *et al. *[33] similarly used GC-MS to demonstrate that serum fatty acid composition differed in a small cohort of Japanese CRC patients compared to healthy controls. Since only 20 patients were examined, it was not feasible to evaluate differences in subgroups. Ludwig *et al. *[34] used NMR spectroscopy to delineate the metabolomic signature of 38 patients with various stages of CRC (including 20 patients with stage IV disease), and identified a typical Warburg signature in association with CRC. The only group so far to specifically study patients with metastatic CRC did not evaluate site of disease as a contributing factor in the metabolomic profile [35]. Moreover, their study population consisted of patients who had been heavily pretreated with multiple cytotoxic chemotherapy regimens. Therefore, the metabolomic profile derived may not be entirely representative of metastatic CRC in general. Interestingly, there were differences in abundance of a number of metabolites between patients who had short survivals and longer survivals. The findings in each of these series will require validation, and further work will be required to evaluate differences in findings in populations from different countries that may occur due to differences in dietary, environmental and genetic factors. Moreover, additional research will be required to identify disease factors that modify the metabolomic signature, including tumor biology, stage and the host response.

One factor that must be further evaluated in the context of our series is the effect of chemotherapy. Patients with metastatic disease were more frequently exposed to chemotherapy within 3 months of sample collection, and it is possible that this influenced our results to some degree. Having said this, there are two lines of evidence that chemotherapy exposure did not have a significant effect. First, regression analysis demonstrated no statistically significant effect on the metabolomic profile. This may be because the time between the last dose of chemotherapy and the date of sample collection was sufficient to 'wash out' any residual metabolic effects of these drugs. Second, we determined that the models derived were unchanged even in individuals who had not received chemotherapy. Ultimately, it will be important to validate our findings in a larger cohort that was not exposed to chemotherapy prior to sample collection.

The finding that metabolomic profile changes with site of disease was surprising and intriguing. The question is whether changes in the circulating metabolites reflect differences in tumor biology or alterations in the host response to tumor, or a combination of both. The host response may change with metastasis because metastatic disease is, by definition, biologically distinct from a cancer that remains confined in the tissue of origin; and more aggressive tumors may incite a more (or less) exuberant response by the host. The response of the host may also differ because of the local effects of tumor. For example, a tumor may have numerous paracrine effects on the surrounding microenvironment, and the metabolic or inflammatory response of surrounding normal tissues may differ between colon, liver and other metastatic sites. The pathway analysis is meant to be hypothesis generating, and this analysis suggested that tumor biology and the host response may both be contributing to the changes in serum metabolomic profile seen with site of disease. Further experimentation on the contributions of various tissues to the circulating metabolome will be required to delineate the relative effects of tumor and host.

In addition to the limitations described above, it is possible that the performance of our metabolomic tests is the result of over-fitting. On the other hand, the generated models demonstrate acceptable and often excellent goodness of fit, as well as satisfactory goodness of prediction for human sample type metabolomic studies. Ultimately, however, it will be imperative to validate our models with a completely independent patient cohort for these metabolites to be useful in a clinical setting.

## Conclusions

We have described a novel observation in which the metabolomic profile of CRC varies with stage and disease site. We must externally validate our findings, to confirm the metabolic profiles observed. This will also aid in determining whether one or both metabolomic analytical modalities (^1^H-NMR spectroscopy and/or GC-MS) will be required to assay for metastatic disease. Further experiments will be required to understand the contributions of tumor and host on the metabolic perturbations in the circulation. Finally, the clinical utility of the tests developed for use in CRC patients will have to be tested in a prospective group of patients being staged for CRC or being followed for recurrence.

## Abbreviations

^1^H NMR: proton nuclear magnetic resonance; AUROC: area under the ROC curve; CRC: colorectal cancer; CT: computed tomography; GC-MS: gas chromatography-mass spectrometry; HMDB: Human Metabolome Database; IL: interleukin; IPA: Ingenuity Pathways Analysis; NF: nuclear factor; O2-PLS-DA: orthogonal partial least squares discriminate analyses (multiple Y components); O-PLS-DA: orthogonal partial least squares discriminate analyses (one Y component); PCA: principal component analysis; ROC: receiver operating characteristic; TNF: tumor necrosis factor.

## Competing interests

The findings described are the subject of a patent application. The authors declare no other competing interests.

## Authors' contributions

FF performed the GC-MS analysis, analyzed all metabolomic data to generate the models, and drafted the manuscript. AMW designed the metabolomic experiments, supervised the metabolomic and pathway analysis, and drafted the manuscript. KK performed the statistical analysis and drafted the manuscript. WDB, AM, ED and FRC provided clinical samples and clinical data, and provided clinical context for the data. AM selected samples for analysis and performed the initial ^1^H-NMR spectroscopy analysis. HJV designed and supervised the ^1^H NMR spectroscopy experiments. OFB conceived of the study, collected clinical samples, and drafted the manuscript. All authors read and approved the final manuscript.

## Supplementary Material

Additional file 1 **Figure S1 - PCA scatter plots of metabolomic profiles**. **(a) **^1^H NMR spectroscopy, locoregional CRC versus liver-only metastases. **(b) **GC-MS spectrometry, locoregional CRC versus liver-only metastases. **(c,d) **Liver-only metastases versus extrahepatic metastases. (c) ^1^H NMR spectroscopy, liver-only metastases versus extrahepatic metastases. (d) GC-MS spectrometry, liver-only metastases versus extrahepatic metastases. t[n], score for the n^th ^principal component in PCA analysis. Click here for file

Additional file 2 **Figure S2 - pathway analysis derived by comparison of the relative abundance of metabolites from sera derived from patients with locoregional CRC and liver-only metastases, as determined by ^1^H NMR spectroscopy**. Click here for file
