# Supplementary figures and images for: Serum metabolomic profile as a means to distinguish stage of colorectal cancer
Source: Genome Med. 2012 May 14;4(5):42. doi: 10.1186/gm341 (PMC3506908; doi:10.1186/gm341)

## Slide 1
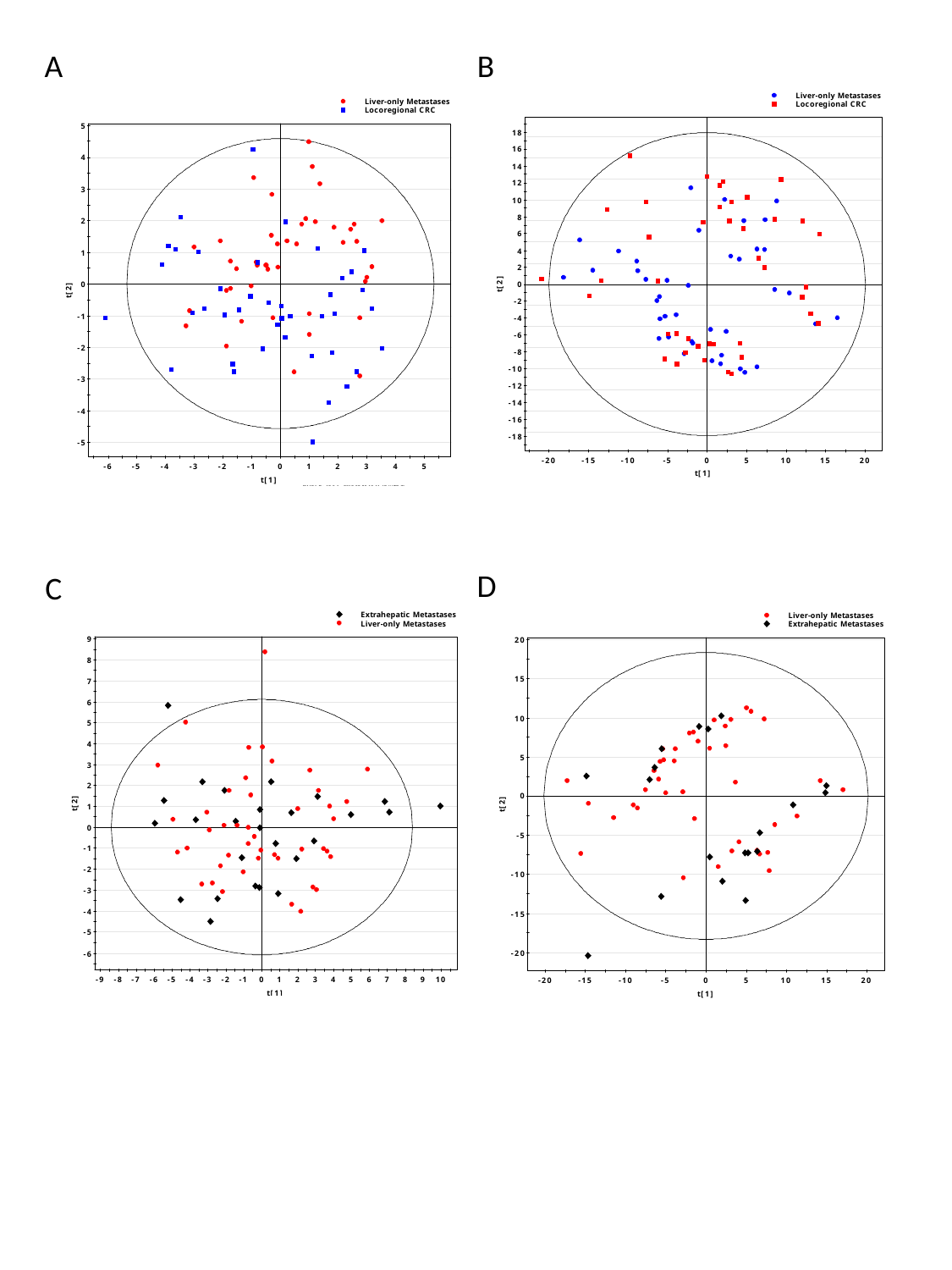

A
B
D
C

Supplement: Additional file 1 — Figure S1 - PCA scatter plots of metabolomic profiles. (a) 1H NMR spectroscopy, locoregional CRC versus liver-only metastases. (b) GC-MS spectrometry, locoregional CRC versus liver-only metastases. (c,d) Liver-only metastases versus extrahepatic metastases. (c) 1H NMR spectroscopy, liver-only metastases versus extrahepatic metastases. (d) GC-MS spectrometry, liver-only metastases versus extrahepatic metastases. t[n], score for the nth principal component in PCA analysis. [file gm341-S1.PPT]

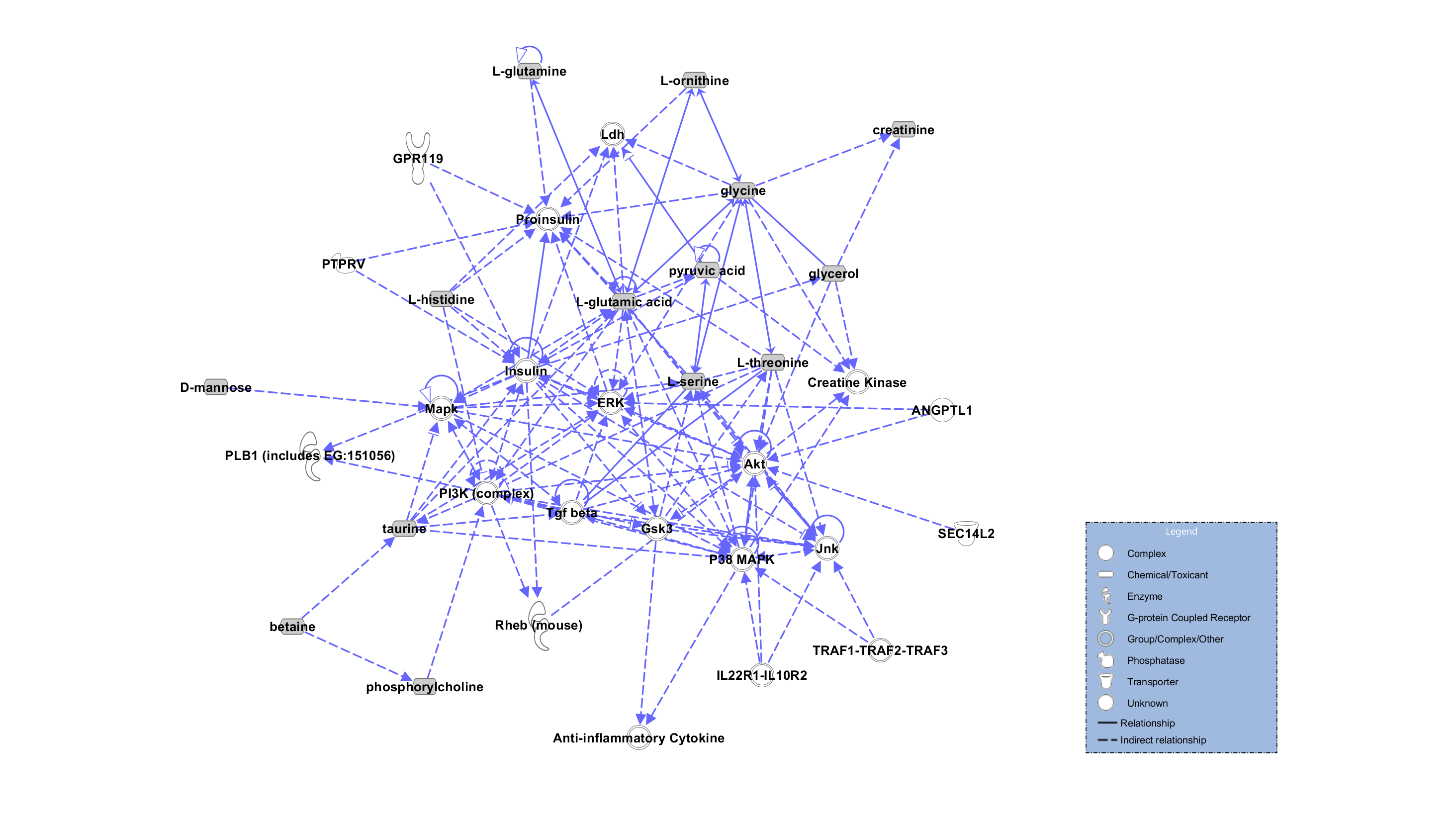

Supplement: Additional file 2 — Figure S2 - pathway analysis derived by comparison of the relative abundance of metabolites from sera derived from patients with locoregional CRC and liver-only metastases, as determined by 1H NMR spectroscopy. [file gm341-S2.TIFF]
